# Real-world evidence for pembrolizumab in non-small cell lung cancer: a nationwide cohort study

**DOI:** 10.1038/s41416-024-02895-1

**Published:** 2024-11-03

**Authors:** Helga H. Hektoen, Kaitlyn M. Tsuruda, Lars Fjellbirkeland, Yngvar Nilssen, Odd Terje Brustugun, Bettina K. Andreassen

**Affiliations:** 1 Department of Research, Cancer Registry of Norway, Norwegian Institute of Public Health, Oslo, Norway; 2 Department of Cancer Genetics, Institute for Cancer Research, Oslo University Hospital, Oslo, Norway; 3 Department of Respiratory Medicine, Oslo University Hospital, Oslo, Norway; 4 Institute of Clinical Medicine, University of Oslo, Oslo, Norway; 5 Department of Registration, Cancer Registry of Norway, Norwegian Institute of Public Health, Oslo, Norway; 6 Section of Oncology, Drammen Hospital, Vestre Viken Health Trust, Drammen, Norway

**Keywords:** Cancer immunotherapy, Non-small-cell lung cancer, Cancer epidemiology

## Abstract

**Background:**

Based on favourable results from clinical trials, immune checkpoint inhibitors (ICI) have become the standard first line (1 L) systemic anticancer treatment (SACT) for advanced stage non-small cell lung cancer (NSCLC) without targetable mutations. We evaluate whether these results are generalizable to everyday clinical practice and compare overall survival (OS) of patients treated with ICI to a historical cohort of patients treated with chemotherapy and results from clinical trials.

**Methods:**

Our study comprised all advanced NSCLC patients initiating SACT in 2012–21 in Norway. Clinical characteristics and treatment information was retrieved from Norwegian Health Registries.

**Results:**

Survival for all 8416 advanced NSCLC patients treated with SACT increased concurrently with the gradual implementation of ICIs. Median OS of patients treated with 1 L pembrolizumab after 2017 was better (mono-/combination therapy: 13.8/12.8 months) than for patients treated with chemotherapy before 2017 (8.0 months). Although median OS for patients treated with pembrolizumab was lower in clinical practice than clinical trials (Keynote-024/189: 26.3/22.0 months), the survival benefit relative to chemotherapy was similar.

**Conclusion:**

Our nationwide study demonstrated a survival benefit over conventional chemotherapy of a similar magnitude as observed in clinical trials and confirms the effectiveness of pembrolizumab in routine clinical practice.

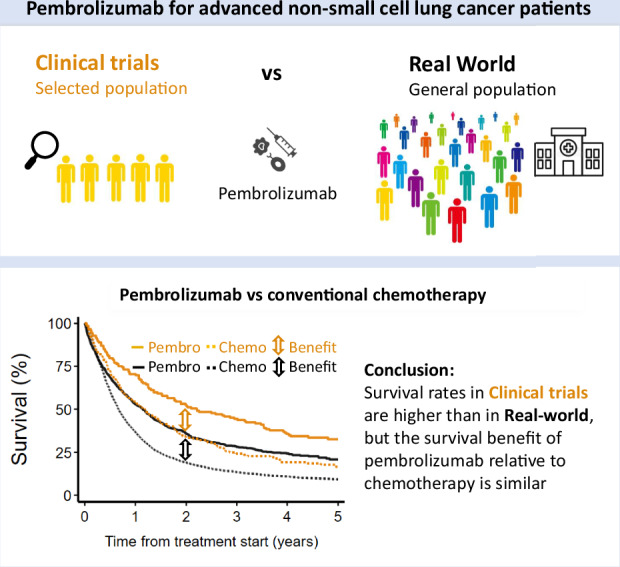

## Background

Non-small cell lung cancer (NSCLC) is often diagnosed at an advanced stage (IIIB to IV) with poor prognosis and historically few treatment options [1, 2]. In recent years, immune checkpoint inhibitors (ICIs) have emerged as the preferred first line (1 L) treatment choice for advanced NSCLC patients not harbouring mutations eligible for targeted therapy, representing a shift away from the previous standard of care, platinum-based chemotherapy [3, 4].

Several ICIs targeting the programmed cell death (PD/PD-L1) pathway have been approved by the US Food and Drug Administration and the European Medicines Agency to treat patients with advanced NSCLC [5]. Pembrolizumab was the first ICI agent to be approved as 1 L treatment (2016) for advanced NSCLC patients without targetable mutations and a PD-L1 expression level ≥50% [4], based on results from the pivotal Keynote-024 trial [6, 7]. This approval was extended (2018/19) as 1 L treatment in combination with platinum-based chemotherapy for these patients (without targetable mutations), regardless of PD-L1 status, first for patients with non-squamous cell histology, supported by results from the Keynote-189 study [8, 9], and later also for those with squamous cell histology, based on results from the Keynote-407 study [10, 11]. Since then, several clinical studies have supported the favourable outcomes of pembrolizumab and other ICIs in the 1 L treatment of advanced NSCLC [12–14].

To validate results from clinical studies conducted on highly selected patients, real-world evidence from a broader patient population treated in routine clinical practice is needed [15, 16]. Recent real-world studies [17–22] have demonstrated promising survival outcomes for NSCLC patients treated with ICIs as 1 L therapy. However, survival estimates vary widely among studies, largely due to heterogeneity in inclusion criteria and sample size. Moreover, these survival estimates have generally been lower than those observed in clinical trials and lack the context of a reference group, making it difficult to interpret the survival benefit associated with ICIs.

To overcome these limitations, we conducted a large, nationwide cohort study with follow-up data until June 30, 2023, comprising all NSCLC patients treated with systemic anticancer treatment (SACT) in Norway from 2012 to 2021. We studied the impact of ICIs, specifically pembrolizumab, in routine clinical practice by comparing overall survival (OS) of patients treated with pembrolizumab (as mono- or combination therapy) to results from both historical chemotherapy and pivotal clinical trials.

## Methods

### Study population

**Fig. 1:**
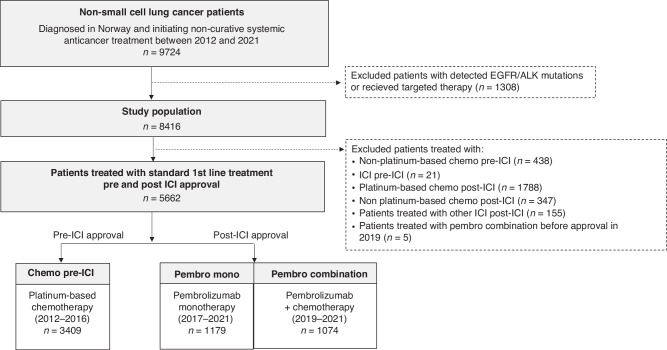
Selection of study population. ICI Immune checkpoint inhibitors, EGFR Epidermal growth factor receptor, ALK Anaplastic large-cell lymphoma kinase, 1 L first line.

This retrospective cohort study included all patients diagnosed with NSCLC as recorded by the Cancer Registry of Norway (CRN) and who initiated non-curative SACT during 2012–21. After excluding patients who had epidermal growth factor receptor (EGFR) mutations or anaplastic large-cell lymphoma kinase (ALK) rearrangements and/or targeted treatment, the study population comprised 8416 patients. Figure 1 illustrates this selection process.

### Data sources

Individual-level data were obtained from three population-based registries: the CRN (including the quality registry for lung cancer, the radiation, and INSPIRE databases) [23, 24], the Norwegian Patient Registry (NPR), and the Norwegian Prescription Database (NorPD). The unique personal identification number assigned to every Norwegian resident facilitated the data linkage for this study.

The CRN provided information about clinical and demographic characteristics related to incident cancer diagnoses such as age, sex, clinical TNM, histology, Eastern Cooperative Oncology Group performance status (ECOG PS), PD-L1 expression, mutation status, vital statistics (including date of death and emigration) and information on radiotherapy. It is mandatory to report cancer cases in Norway and the CRN has thus a high level of completeness for lung cancer cases (99%) [24]. Detailed information on SACT was obtained from the INSPIRE database, which represented nearly 90% of patients treated in Norway in 2019 [23]. Complementary information on SACT was retrieved from NPR [25].

Information about autoimmune disorders was obtained from NPR and NorPD. Patients were classified as having a recent history of an *autoimmune disease* if registered in NPR or NorPD up to 2 years prior to initiating SACT with an ICD-10 or ICPC-2 code indicating at least one disease in the following categories: rheumatologic, dermatologic, endocrine, gastrointestinal, or neurologic.

Information about prescription drugs, including targeted treatment, was obtained from the NorPD.

Information about education and household income was obtained from Statistics Norway. Education was categorized in two categories after the highest obtained educational level, and income was categorized in three categories after household income the year before diagnosis (low; bottom 30%, intermediate; middle 40%, or high; top 30%).

### Identification of anticancer treatment

In this study, SACT was defined as 1) ICI, including pembrolizumab, nivolumab, or atezolizumab; or as 2) any chemotherapies given to advanced NSCLC patients, as verified by an oncologist (Supplementary Table A.1). Chemotherapies were further categorized as platinum-based (if containing cisplatin or carboplatin) or not. Chemotherapy given in a neoadjuvant/adjuvant setting with surgery or concomitantly with radiotherapy (i.e. chemoradiotherapy) was assumed to be given with a curative intent and was not categorized as SACT.

*Palliative radiation* was defined based on the intention to treat information provided by the oncologist and registered in the radiation information system. Radiation against the brain was defined based on specific region codes. Patients were classified as having *active brain metastases* if they received radiation to the brain within ±1 month of initiating SACT.

*First line (1* *L)* SACT was defined as an initial systemic treatment between 2012 and 2021 after an initial advanced diagnosis (stage IIIB-C/IV) or as the first registered non-curative SACT in patients progressing from local disease. Palliative radiation did not count when defining treatment lines.

### Real world treatment

Cancer therapy in Norway is provided by the public health care system after approval by the Norwegian Medicines Agency and thus accessible for all Norwegians.

We separated patients into three treatment groups (Fig. 1), reflecting the standard 1 L treatment in Norway within different treatment periods.

- The *chemo pre-ICI* group consisted of patients initiating 1 L platinum-based chemotherapy from 2012 through 2016, i.e. before 1 L ICI was available.
- The *pembro mono* group consisted of patients initiating 1 L pembrolizumab following pembrolizumab approval as monotherapy from 2017 through 2021.
- The *pembro combination* group consisted of patients initiating 1 L pembrolizumab in combination with chemotherapy from 2019 when combination therapy was first approved for patients with non-squamous cell histology (includes patients diagnosed with squamous cell histology during 2020–2021).

To account for differences in patient characteristics when comparing OS between these groups, we used raked weights to adjust the chemo group for sex, age (<60, 60–64, 65–69, 70–74, ≥75), histology (squamous, non-squamous), and stage to better reflect the patient characteristics of the respective pembro groups.

### Comparison to clinical trials

We compared observed OS with the results of two clinical trials: Keynote-024 for monotherapy and Keynote-189 for combination therapy. Of note, Keynote-189 solely included patients with non-squamous cell histology, however, most patients receiving the combination therapy in our study population had non-squamous cell histology. For better comparison with these trial results, we matched our study population to the trial population using the selection criteria outlined in the respective clinical trials [6, 8] based on available variables, including ECOG PS, stage, autoimmune disease, brain metastasis, PD-L1 status and histology. Additionally, we applied raked weights such that the marginal distributions of sex, age (<65 years, ≥65 years), and histology (pembrolizumab monotherapy group only) reflected those in the respective trial.

### Statistical analyses

Baseline patient and clinical characteristics were presented using descriptive statistics. Means with standard deviation (SD) or medians with range were reported for continuous variables. Frequencies with percentages were presented otherwise.

The primary outcome was OS estimated using the Kaplan-Meier method. Patients were followed from initiation of first SACT until death. Follow-up time was censored for patients who emigrated before or were alive at the end of the study period (June 30, 2023). Median potential follow-up time calculated using the reverse Kaplan-Meier estimator was 101, 48, and 31 months for the chemo pre-ICI, pembro mono, and pembro combination groups, respectively [26]. Within these groups, there were 3203, 891, and 786 deaths from any cause.

To compare our results to the Kaplan-Meier curves from the selected Keynote studies we used a method and algorithm described by Guyot et al. to reconstruct the published survival curves using WebPlotDigitizer [27] and an R script provided by Guyot et al. [28]. These comparisons were descriptive only and no statistical testing was performed.

The survival benefit of pembrolizumab mono- or combination therapy was defined as absolute improvement in survival in percentage points (%pt) when compared to chemotherapy treatment.

Raked weights were calibrated using the ipfraking package [29] and differences in survival probabilities between Kaplan-Meier survival curves (ICI versus chemo) were estimated using the stsurvdiff package [30]. Statistical analyses were performed using Stata version 18.5 unless otherwise mentioned. For analyses in R, version 3.5.3 was used.

## Results

### Patient characteristics

The clinical characteristics of the 8416 advanced NSCLC patients who initiated non-curative SACT between 2012 and 2021, overall and stratified by three treatment periods, are presented in Table 1. Median age at treatment start was 69 years, increasing from 67 years in the pre-ICI era (2012–16) to 70 years in the last time interval (2019–21). Over half (56%) of patients were male, and the predominant histological subtype was non-squamous cells (75%). Two-thirds of treated patients were diagnosed with a primary advanced IIIB/C or IV, and about one-third had progressed from a non-advanced disease. Moreover, 79% of patients with available ECOG PS had a score of 0–1. This proportion decreased over the study period (pre-ICI: 82% to 77% in 2019–21).

**Table 1:** Patient characteristics for all advanced non-small cell lung cancer patients treated with 1 L pembrolizumab mono- or combination therapy in Norway (real-world) or clinical trials (Keynote-024 and -189) and platinum-based chemotherapy in the pre-ICI era (real-world).

	All	Pre-ICI	ICI	
Treatment initiation	2012–21	2012–16	2017–18	2019–21
Patients, *N*	*N* = 8416	*N* = 3868	*N* = 1862	*N* = 2686
Age at treatment start, median [range]	69 (22,93)	67 (22,92)	69 (28,91)	70 (36,93)
Sex				
Female	3693 (44%)	1660 (43%)	806 (43%)	1227 (46%)
Male	4723 (56%)	2208 (57%)	1056 (57%)	1459 (54%)
Histology				
Squamous	2103 (25%)	933 (24%)	448 (24%)	722 (27%)
Non-squamous	6313 (75%)	2935 (76%)	1414 (76%)	1964 (73%)
Stage at treatment start				
IIIB/C	744 (9%)	299 (8%)	165 (9%)	280 (10%)
IV	4747 (56%)	2208 (57%)	1006 (54%)	1533 (57%)
Progressed from I-IIIA	2925 (35%)	1361 (35%)	691 (37%)	873 (33%)
ECOG-PS				
Known	4704	1001	1387	2316
0–1	3720 (79%)	819 (82%)	1106 (80%)	1795 (78%)
2–4	984 (21%)	182 (18%)	281 (20%)	521 (22%)
Unknown	3712 (44%)	2867 (74%)	475 (26%)	370 (14%)
History of autoimmune disease				
No	6435 (77%)	3076 (80%)	1391 (75%)	1968 (73%)
Yes	1981 (23%)	792 (20%)	471 (25%)	718 (27%)
1 L systemic anticancer treatment				
Platinum-based chemotherapy	5197 (62%)	3409 (88%)	1109 (60%)	679 (25%)
Non-platinum-based chemotherapy	785 (9%)	438 (11%)	258 (14%)	89 (3%)
Pembrolizumab monotherapy	1192 (14%)	13 (0.3%)	416 (22%)	763 (28%)
Pembrolizumab combination	1079 (13%)	0 (0.0%)	5 (0.3%)	1074 (40%)
Other ICI (atezolizumab, nivolumab)	163 (2%)	8 (0.2%)	74 (4%)	81 (3%)
*Additional palliative treatment*				
Palliative radiation				
No	4604 (55%)	1869 (48%)	1018 (55%)	1717 (64%)
Yes	3812 (45%)	1999 (52%)	844 (45%)	969 (36%)
Brain radiation				
No	7289 (87%)	3356 (87%)	1557 (84%)	2376 (89%)
Yes	1127 (13%)	512 (13%)	305 (16%)	310 (11%)
*Socioeconomic status*				
Household income year before diagnosis				
Low	3524 (42%)	1631 (42%)	755 (41%)	1138 (42%)
Intermediate	3579 (43%)	1611 (42%)	813 (44%)	1155 (43%)
High	1227 (15%)	578 (15%)	277 (15%)	372 (14%)
Missing	86 (1%)	48 (1%)	17 (1%)	21 (1%)
Highest obtained education				
Vocational school, high school, or lower	7254 (86%)	3370 (87%)	1608 (86%)	2276 (85%)
College or university	1091 (13%)	460 (12%)	243 (13%)	388 (14%)
Missing	71 (1%)	38 (1%)	11 (1%)	22 (1%)

Data are presented as frequency (%) unless otherwise indicated.

*ECOG PS* Eastern Cooperative Oncology Group performance status, *1 L* first line, *ICI* immune checkpoint inhibitors.

### Overall systemic anticancer treatment and survival trends in Norway from 2012–21

**Fig. 2:**
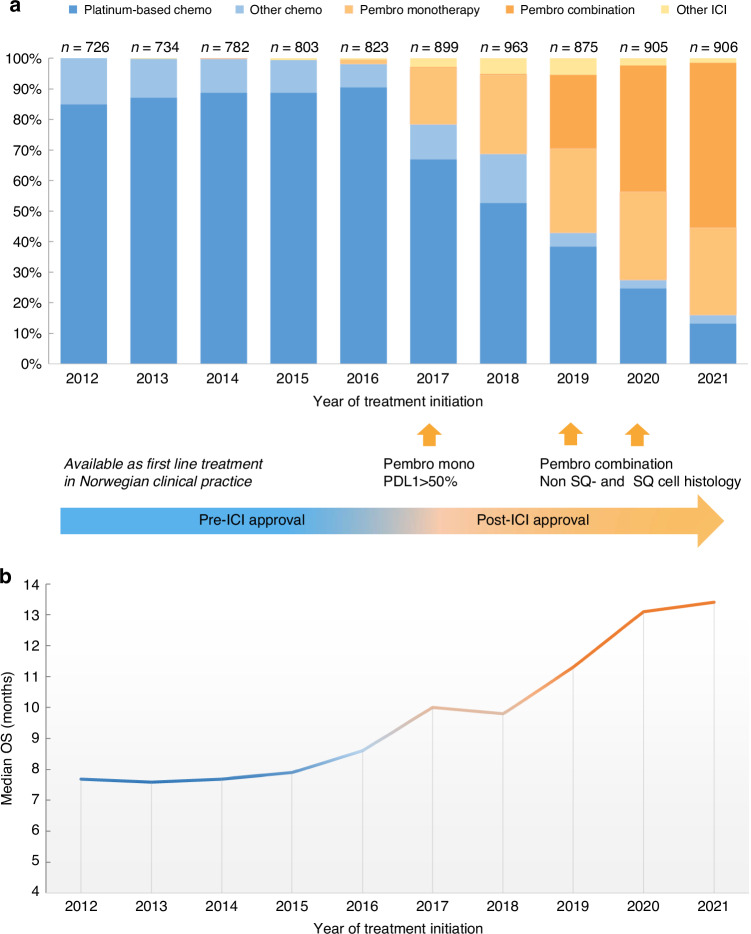
First-line systemic treatment trends and survival. Trends in first-line systemic anticancer treatment for advanced non-small cell lung cancer patients in Norway from 2012–2021, displaying (**a**) patterns of first-line treatment and (**b**) median overall survival (OS) of these patients by year of treatment initiation. ICI Immune check point inhibitors; OS Overall survival; SQ Squamous cell; PDL1 programmed death ligand 1.

The annual number of patients receiving SACT increased from 726 to 906 between 2021 and 2021 (Fig. 2A and Supplementary Table A.2). Most patients (85–90%) in the pre-ICI era received platinum-based chemotherapy in 1 L in clinical practice. Thereafter, there was a gradual increase in patients receiving 1 L treatment with ICIs, from 19% in 2017 to 84% in 2021. More specifically, in the first years following approvals, most patients treated with 1 L ICI received pembrolizumab as monotherapy (19% in 2017 to 26–29% in 2018–21), and from 2019, pembrolizumab in combination with chemotherapy (2019: 24%, 2020: 41%, 2021: 54%). A few patients (2–5%) received other ICI drugs (1 L), including nivolumab and atezolizumab.

Median survival increased from about 8 months (between 7.6 and 8.6 months) for patients treated with advanced SACT in the pre-ICI era to 10.0 months in 2017 and 13.4 months in 2021 (Fig. 2B and Supplementary Table A.2).

### 1 L Pembrolizumab as mono- or combination therapy versus platinum-based chemotherapy (pre-ICI era)

Patients receiving platinum-based chemotherapy as 1 L treatment in the pre-ICI era (*n* = 3409) were younger (median 67 years) than patients receiving pembrolizumab as monotherapy (median 71 years; *n* = 1179), or in combination with chemotherapy (median 69 years; n = 1074) (Table 2). Additionally, more women received pembrolizumab (mono- and combination therapy) than chemotherapy (47% and 48% vs 43%), and more patients with high ECOG status received pembrolizumab monotherapy than chemotherapy alone or in combination with pembrolizumab (27% vs 19% and 20%).

**Table 2:** Patient characteristics for all advanced non-small cell lung cancer patients treated with systemic treatment in Norway from 2012–21, both overall and stratified for three different time periods corresponding to available treatments.

	Chemo-pre ICI	Pembro mono		Pembro combination	
	*Real-world*	*Real-world*	*Keynote-024*	*Real-World*	*Keynote-189*
Patients, *N*	*N* = 3409	*N* = 1179	*N* = 154	*N* = 1074	*N* = 410
Age, median [range]	67 (28,92)	71 (32,93)	65 (33,90)	69 (36,87)	65 (34,84)
Age, categories					
<65 years	1237 (36%)	295 (25%)	71 (46%)	328 (31%)	197 (48%)
65–75 years	1551 (46%)	491 (42%)	60 (39%)	517 (48%)	213 (52%)^a^
>75 years	621 (18%)	393 (33%)	22 (15%)	229 (21%)	–
Sex					
Females	1451 (43%)	562 (48%)	62 (40%)	505 (47%)	156 (38%)
Males	1958 (57%)	617 (52%)	92 (60%)	569 (53%)	254 (62%)
Histology					
Squamous	820 (24%)	328 (28%)	29 (19%)	156 (14%)	–
Non-squamous	2589 (76%)	851 (72%)	125 (81%)	918 (86%)	410 (100%)
Stage					
IIIB/C	255 (8%)	131 (11%)	–	102 (10%)	-
IV	2030 (59%)	705 (60%)	154 (100%)	745 (69%)	410 (100%)
Progressed from I-IIIA	1124 (33%)	343 (29%)	–	227 (21%)	–
ECOG PS					
Known	934	1000	154	952	407
0–1	760 (81%)	727 (73%)	54 (99%)	757 (80%)	406 (>99%)
2–4	174 (19%)	273 (27%)	1 (1%)	195 (20%)	1 (<1%)
Unknown	2475 (73%)	179 (15%)		122 (11%)	3 (1%)
History of autoimmune disease					
No	2721 (80%)	873 (74%)	154 (100%)	797 (74%)	406(>99%)
Yes	688 (20%)	306 (26%)		277 (26%)	1 (<1%)
Active brain metastases					
No	–	1116 (95%)	154 (100%)	1029 (96%)	410 (100%)
Yes	–	63 (5%)	–	45 (4%)	–
PD-L1 expression level					
<1%	75 (2%)	26 (2%)	–	332 (31%)	127(31%)
1–49%	103 (3%)	67 (6%)	–	305 (28%)	128(31%)
>50%	88 (3%)	979 (83%)	154 (100%)	185 (17%)	132(32%)
Unknown result	33 (1%)	52 (4.4%)	–	195 (18%)	23 (6%)
Not tested	3110 (91%)	55 (4.7%)	–	57 (5.3%)	–

Data are presented as frequency (%) unless otherwise indicated.

*ECOG PS* Eastern Cooperative Oncology Group performance status, *1* *L* first line, *PD-L1* programmed death ligand 1, *ICI* immune checkpoint inhibitors.

^a^Includes all patients ≥ 65 years.

**Fig. 3:**
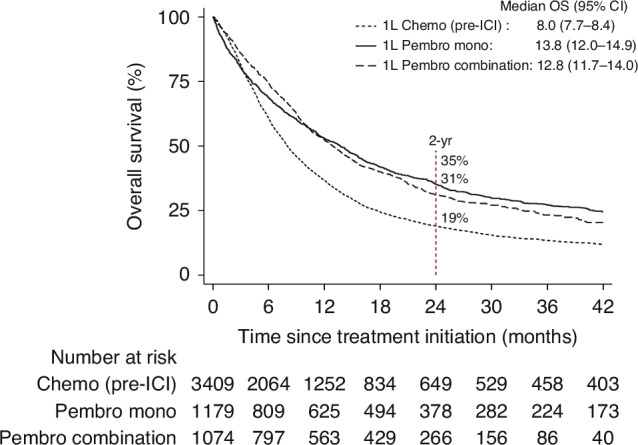
Overall survival: pembrolizumab vs chemotherapy. Overall survival for advanced non-small cell lung cancer patients treated with first line(1 L) pembrolizumab (pembro mono- and combination therapy) compared to 1 L platinum-based chemotherapy in the period before immune checkpoint inhibitors (ICI) were available (Chemo-pre-ICI). The plot shows median overall survival (OS) with 95% confidence interval (CI) and estimated 2-year (2-yr) OS, for the respective treatment groups. ICI Immune check point inhibitors; OS Overall survival; CI confidence interval

Median OS was better for patients treated with 1 L pembrolizumab, both mono- and combination therapy, compared to platinum-based chemotherapy (median OS 13.8 and 12.8 vs 8.0 months; Fig. 3). Two-year OS was also higher for patients receiving pembrolizumab as mono- or combination therapy than for those receiving platinum-based chemotherapy (35% and 31% vs 19%). During the 4–5 months following treatment initiation, the survival probabilities between treatment groups were similar. The survival probabilities diverged thereafter. The survival differences between patients treated with pembrolizumab versus chemotherapy were similar after adjusting for differences in the patient characteristics (Supplementary Fig. A.1).

### 1 L Pembrolizumab as mono- or combination therapy in clinical practice in Norway versus clinical trials

#### Pembrolizumab monotherapy in real-world vs Keynote-024

Compared to patients receiving 1 L pembrolizumab monotherapy in clinical practice in Norway from 2017–21, patients included in the Keynote-024 clinical trial were younger (median age 65 vs 71 years), a larger proportion were men (60 vs 52%), and a higher proportion was diagnosed with a non-squamous cell histology (81 vs 72%; Table 2). Additionally, certain patient characteristics that were excluded from the Keynote-024 trial were represented within the real-world population, including patients who had ECOG PS > 1 (27%), a history of autoimmune disease(s) (12%), active brain metastases (5%), a PD-L1 expression below 50% (17%), or stage IIIB/C disease at diagnosis (11%).

**Fig. 4:**
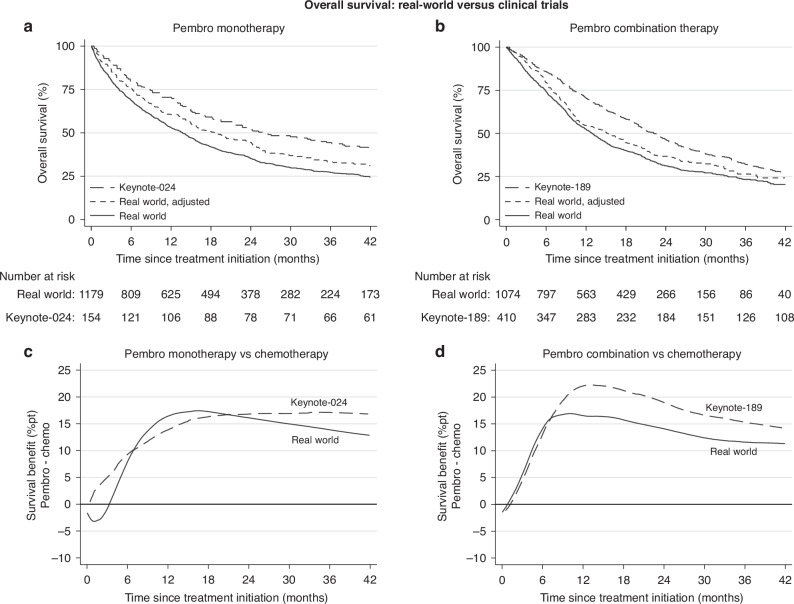
Overall survival in real-world vs clinical trials. Pembrolizumab (Pembro) as mono- or combination therapy in clinical practice in Norway versus clinical trials, comparing overall survival (**a**, **b**) and survival benefits over chemotherapy (Chemo) (**c**, **d**). Survival benefit is defined as the absolute difference (in %pt) between the survival curve of the respective ICI treatment group and the survival curve of chemotherapy treatment group (pembro—chemo). A survival benefit (difference) of zero means that there is no survival gain of 1 L pembrolizumab compared to chemotherapy treatment. A positive survival benefit is equivalent to a survival gain of 1 L pembrolizumab compared to chemotherapy treatment.

Median OS was 13.8 months among all patients treated with 1 L pembrolizumab monotherapy compared to 26.3 months for those included in the Keynote-024 trial (Fig. 4A). After adjusting the patient characteristics of the real-world population to better reflect those in the Keynote-024 population, median OS increased to 18.9 months. Importantly, the treatment benefit (pembrolizumab vs chemotherapy, Fig. 4C) was similar in the real-world and Keynote populations with an absolute increase in 2-year survival of 17%pt in both: from 19% to 35% in the real-world population and from 34% to 51% in the Keynote population. These survival benefits first became visible in the real-world population 4-6 months after initiating treatment.

#### Pembrolizumab combination therapy in real world vs Keynote-189

Compared to the real-world population, patients enrolled in the Keynote-189 study were younger (median age 65 vs 69 years) and a larger proportion were men (62 vs 53%). Moreover, all Keynote-189 patients had non-squamous cell histology compared to 86% in the real-world population. Again, certain patient characteristics that were excluded from the Keynote-189 trial were represented within the real-world population receiving pembrolizumab combination therapy, including patients with squamous cell histology (14%), patients who had an ECOG PS > 1 (18%), a history of autoimmune disease(s) (12%), active brain metastasis at treatment start (4%) or stage IIIB/C disease at diagnosis (10%).

Median OS after initiating 1 L pembrolizumab combination therapy was lower for real-world patients in Norway than those included in the Keynote-189 trial (12.8 vs 22.0 months; Fig. 4B). After adjusting the patient characteristics of the real-world population to match the Keynote-189 population, median OS increased to 15.2 months. Moreover, the treatment benefit (pembrolizumab combination therapy vs chemotherapy; Fig. 4D) was similar between the real-world and Keynote populations up to 8 months after treatment initiation; the survival benefit was more pronounced in the keynote population thereafter. The absolute increase in 2-year survival was 12%pt (from 19% to 31%) in the real-world population and 18%pt (from 28% to 46%) in the Keynote study.

## Discussion

In this comprehensive nationwide real-world study, we followed all advanced NSCLC patients treated with SACT from 2012 through 2021 in Norway and observed a significant increase in survival as expected due to the introduction of ICIs. We also observed improved survival among patients receiving 1 L pembrolizumab, either as monotherapy or in combination with chemotherapy, compared to those receiving platinum-based chemotherapy before ICI approval. The probability of survival for patients treated in clinical practice in Norway was not as advantageous as that reported in clinical trials, even after adjusting for differences in patient characteristics. However, the survival benefits of pembrolizumab over platinum-based chemotherapy were similar in our real-world study compared to clinical trial results.

### Other real-world studies

We observed a median OS of 13.8 months in patients treated with 1 L pembrolizumab as monotherapy and 12.8 months when given in combination with chemotherapy. The median OS reported in other real-world studies ranges between 10–23 months [17–22, 31]. In particular, our results are comparable to another large real-world study [17] of over 3000 patients from the Flatiron Health database treated with monotherapy (median OS 10–11 months), and over 4200 patients receiving combination therapy (median OS 10-14 months). Another comparable multicenter French cohort study reported a rather high median OS of 22.6 months for patients treated with 1 L pembrolizumab monotherapy in a real-life clinical setting [20]. However, their patient cohort differed from ours by only including patients with PD-L1 expression level >50% and having a somewhat higher proportion of patients with good(0-1) ECOG PS (78 vs 73% in our cohort). Moreover, their patient population was younger (median age 65 years vs our study’s 71 years). The higher median OS in this French multicenter study might therefore partly be explained by having included more patients associated with a better initial prognosis. There are several other population-based studies that report higher median OS estimates than we observed [19, 21, 22, 31]. In general, these observational studies were not nationwide, had smaller sample sizes and were often restricted to patients with ECOG PS 0 or 1, and/or PD-L1 scores ≥ 50%, which probably led to improved patient outcomes. This makes the generalizability of results from these observational studies to *all* patients treated with ICIs in general clinical practice questionable.

### Survival trends pre and post ICI approval

Before ICI approval, the median OS was relatively stable at approximately 8 months, which is similar to that reported for advanced patients (stage IIIB to IV) treated with platinum-based chemotherapy in the pre-ICI era (median OS range 8–10 months) [32]. Similar to a previous Norwegian publication, we observed an improvement in median OS following the introduction of ICI in 2017 [33]. We note that we did observe a slight improvement in survival already in 2016—this could be due to some patients participating in clinical trials prior to the general approval or from patients benefiting from second or later-line ICI treatment.

### Comparison to pre-ICI chemotherapy

When comparing patients treated with 1 L pembrolizumab (as monotherapy or in combination with chemotherapy) to patients treated with platinum-based chemotherapy before the introduction of ICIs in Norway, we observed a 5–6-months improvement in median OS from 8.0 up to 13.8 and 12.8 months and a nearly doubled 2-year OS from 19% up to 35% and 31%, respectively. A Danish study [18] reported a median OS increase of 9 months (from 10 months to 19 months) when comparing patients receiving 1 L ICIs (monotherapy) to a historical chemotherapy group. This exceeded the improvement in median survival that we observed, even though their historical comparison cohort had a better prognosis. However, the Danish ICI cohort included a lower proportion of patients with ECOG PS 2 (15% vs 23% in our cohort) and a larger proportion of patients with PD-L1 > 50% (95% vs 83%), which might at least partly explain their favourable results. Additionally, similar to our findings, they also observed a doubling in estimated 2-year OS (from 22 to 42%) over chemotherapy.

When interpreting these results, we need to take into consideration that a more “liberal” treatment culture for older and more fragile patients has evolved in later years, as these patients seem to tolerate the side effects of ICI better than chemotherapy [34]. We observed that patients treated with pembrolizumab were older and had more comorbidities than patients treated with chemotherapy in the pre-ICI era. However, adjusting the survival curves with respect to differences in patient characteristics produced similar results.

### Comparison to clinical trial results

Median OS for patients receiving pembrolizumab in clinical practice in Norway, both as monotherapy (13.8 months) and in combination with chemotherapy (12.8 months), was about a year shorter than the respective pembrolizumab arm in the Keynote studies used for comparison (26.3 months and 22.0 months, respectively) [7, 9]. The observed survival improved after adjusting our study populations to resemble the respective Keynote populations more closely, but these adjusted estimates remained lower than those reported in the Keynote trials and other similar clinical studies [10, 12]. Cross-trial comparisons between clinical trials and real-world data are challenging, particularly due to the lack of key clinical information in real-world data. Furthermore, there are general differences between patients recruited in clinical trials and treated in clinical practice such as that patients recruited to clinical trials generally are healthier, have more stable disease, and receive more comprehensive follow-up than those treated in everyday clinical practice [35]. Thus, although we were able to adjust for most critical clinical variables, these challenges complicate the presented comparisons to clinical trial results. However, comparing survival benefits (within each study type) and the use of a historical cohort (comparison to pre-ICI chemotherapy, Section 4.3) to obtain within-population comparisons offer valuable approaches to address these challenges. Despite lower survival probabilities reported in our study than in clinical trials, the survival benefit of pembrolizumab monotherapy (vs chemotherapy) in our study was similar to that reported in clinical trials. This benefit did not start at treatment initiation in our study, however, this is in line with another real-world study which also observed a 5-6 month lag in the survival benefit of pembrolizumab monotherapy [36]. The poorer medical condition of some patients treated with ICIs in the real world may partially explain this finding. For patients receiving pembrolizumab combination therapy, the survival benefit relative to chemotherapy was similar between the real-world and Keynote population the first months after treatment initiation and in favour of the Keynote population thereafter. This might at least partly be explained by less favourable outcomes in the Keynote-189 chemotherapy arm compared to other previous studies [8]. Moreover, our real-world study population also included patients with squamous cell histology (14%), who were excluded from the Keynote-189 trial. The Keynote-407 trial (which led to approval for treating squamous cell NSCLC) showed only 5%pt survival benefits after 2 years (36% vs 31%) for patients treated with combination therapy over chemotherapy, compared to 19%pt in the Keynote-189 trial (46% vs 27%), suggesting that these patients benefit less from 1 L ICI and this could have affected our results [10, 11].

### Limitations

This study had several limitations. As this was an observational study, some clinical information was not available or was inconsistently collected. For example, ECOG PS was largely missing in the pre-ICI approval period. We also lacked detailed information on relapse, which precluded us from reporting progression-free survival. We cannot rule out that we might have included some patients with EGFR/ALK mutations, as not all patients were tested for mutations. However, as we had detailed information about targeted therapy, we assume that we identified most of the cases that were not eligible for this study.

It is essential to acknowledge the potential influence of improved diagnostics and refined treatment management over time when comparing the survival benefits of new treatments to the previous standard of care in observational studies [37]. However, since patients in our study were treated within a very short time frame, we assume that these improvements had little influence on survival outcomes in our study.

### Strengths

The main strengths of this study are that we included virtually *all* advanced NSCLC patients treated with SACT in Norway over the period of ICI introduction (2012–21), allowing us to study an unselected population treated in everyday clinical practice. This was made possible by Norway’s publicly financed healthcare system, which provides equal treatment to all, and where cancer reporting is mandatory. Another strength of this study is that we employed a large historical comparison cohort reflecting the patient population eligible for SACT before the introduction of ICIs. This allowed us to evaluate the survival gain of ICIs over previous standard treatment within a real-world population.

## Conclusion

The OS of advanced NSCLC patients treated in everyday practice in Norway has improved since ICIs became available as 1 L treatment in 2017. When comparing improvement in survival with pembrolizumab relative to previous platinum-based chemotherapy, we observed a doubling in patients surviving more than 2 years. Although the survival outcomes were not as high as reported in pivotal clinical trials, the survival benefit relative to chemotherapy was similar. Future research is necessary to better understand which patients benefit from ICIs in clinical practice and which do not.

## Supplementary information

Reproducibility Checklist

Appendix

STROBE checklist

## Data Availability

The Cancer Registry of Norway’s data-delivery unit handles all inquiries about data from the Cancer Registry of Norway via application to Helsedata.no. Access will be conditional to adherence to local ethical and security policy.

## Code availability

The code used to conduct specific analyses is available from the authors upon request.
