# Supplementary material for: Real-world evidence for pembrolizumab in non-small cell lung cancer: a nationwide cohort study
Source: Br J Cancer. 2024 Nov 3;132(1):93–102. doi: 10.1038/s41416-024-02895-1 (PMC11724112; doi:10.1038/s41416-024-02895-1)
Supplement: Supplementary file 2 — Appendix [file 41416_2024_2895_MOESM2_ESM.docx]

***Appendix A – Supplementary tables and figures***

**Supplementary Figure A.1** Overall survival for advanced non-small cell lung cancer patients treated with first line(1L) pembrolizumab (pembro mono- and combination therapy) both unadjusted and adjusted for age, stage, histology and sex compared to 1L platinum-based chemotherapy in the period before immune checkpoint inhibitors (ICI) were available (Chemo-pre-ICI).


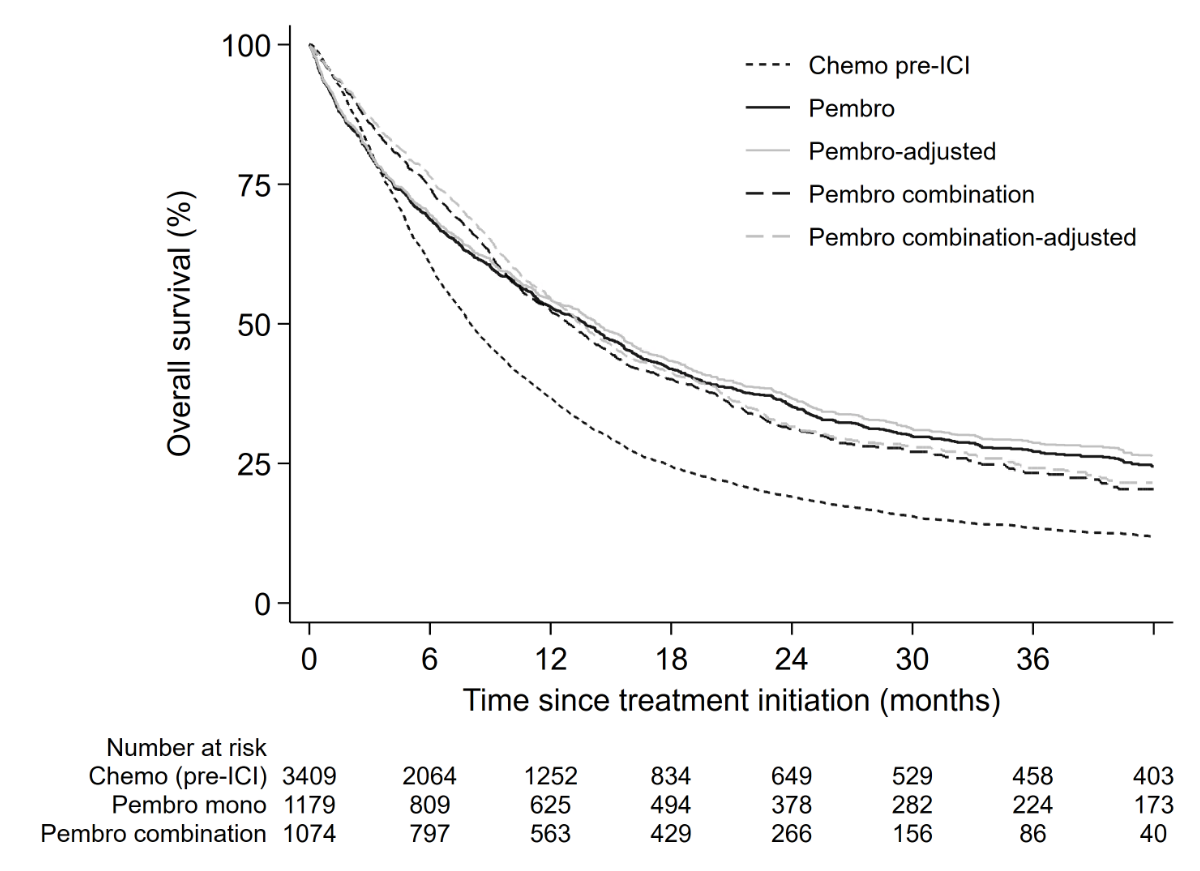


**Supplementary Table A.1**: Type of chemotherapy-agents given to advanced NSCLC patients in Norway from 2012-21

| **First line - platinum-based chemotherapy** |
| --- |
| **Carboplatin** as single agent or in combinations with one or more of the following agents: vinorelbine, pemetrexed, etoposide, gemcitabine, paclitaxel and bevacizumab |
| **Cisplatin** as single agent or in combinations with one or more of the following agents: vinorelbine, etoposide or gemcitabine |
| **First line - other chemotherapies** |
| One or more of the following (either as single agent or in combinations): vinorelbine, pemetrexed, paclitaxel, docetaxel, etoposide or gemcitabine |
| **First line- pembrolizumab in combination with chemotherapy** |
| Pembrolizumab in combinations with carboplatin and (pemetrexed or paclitaxel) |

**Supplementary Table A.2**  Median overall survival (OS) in months, including 95% confidence intervals (95%CI) for non-small cell lung cancer patients stratified by year of treatment initiation; and number of patients for each type of first-line systemic anticancer treatment given, stratified by year.

|  | **2012** | **2013** | **2014** | **2015** | **2016** | **2017** | **2018** | **2019** | **2020** | **2021** |
| --- | --- | --- | --- | --- | --- | --- | --- | --- | --- | --- |
| Overall survival (OS) | | | | | | | | | | |
| Median OS (95%CI) | 7.7  (6.9-8.8) | 7.6  (7.0-8.4) | 7.7  (6.9-8.5) | 7.9  (7.5-8.5) | 8.6  (8.0-9.7) | 10.0  (8.6-11.1) | 9.8  (8.9-10.7) | 11.3  (10.0-12.9) | 13.1  (11.7-14.5) | 13.3  (12.0-14.8) |
| First-line treatment | | | | | | | | | | |
| Platinum-based chemo | 617 (85.0%) | 640 (87.2%) | 694 (88.7%) | 713 (88.8%) | 745 (90.5%) | 602 (67.0%) | 507 (52.6%) | 336 (38.4%) | 223 (24.6%) | 120 (13.2%) |
| Other chemo | 109 (15.0%) | 93 (12.7%) | 87 (11.1%) | 86 (10.7%) | 63 (7.7%) | 103 (11.5%) | 155 (16.1%) | 39 (4.5%) | 25 (2.8%) | 25 (2.8%) |
| Pembro mono | 0 (0.0%) | 0 (0.0%) | 1 (0.1%) | 0 (0.0%) | 12 (1.5%) | 167 (18.6%) | 249 (25.9%) | 242 (27.7%) | 262 (29.0%) | 259 (28.6%) |
| Pembro combination | 0 (0.0%) | 0 (0.0%) | 0 (0.0%) | 0 (0.0%) | 0 (0.0%) | 2 (0.2%) | 3 (0.3%) | 211 (24.1%) | 374 (41.3%) | 489 (54.0%) |
| Other ICIs | 0 (0.0%) | 1 (0.1%) | 0 (0.0%) | 4 (0.5%) | 3 (0.4%) | 25 (2.8%) | 49 (5.1%) | 47 (5.4%) | 21 (2.3%) | 13 (1.4%) |
| Total | 726 | 734 | 782 | 803 | 823 | 899 | 963 | 875 | 905 | 906 |
